# Medicinal Polypharmacology in the Clinic – Translating the Polypharmacolome into Therapeutic Benefit

**DOI:** 10.1007/s11095-024-03656-8

**Published:** 2024-02-16

**Authors:** Muhammad Rafehi, Marius Möller, Wouroud Ismail Al-Khalil, Sven Marcel Stefan

**Affiliations:** 1 Department of Medical Education Augsburg, Augsburg University Medicine, Stenglinstr. 2, 86156 Augsburg, Germany; 2 Institute of Clinical Pharmacology, University Medical Center Göttingen, Robert-Koch-Str. 40, 37075 Göttingen, Germany; 3 Medical Systems Biology Group, Lübeck Institute of Experimental Dermatology (LIED), University of Lübeck and University Medical Center Schleswig-Holstein, Ratzeburger Allee 160, 23538 Lübeck, Germany; 4 Medicinal Chemistry and Systems Polypharmacology, Medical Systems Biology Division, Lübeck Institute of Experimental Dermatology (LIED), University of Lübeck and University Medical Center Schleswig-Holstein, Ratzeburger Allee 160, 23538 Lübeck, Germany; 5 Department of Pathology, University of Oslo and Oslo University Hospital, Sognsvannsveien 20, 0372 Oslo, Norway; 6 Department of Biopharmacy, Medical University of Lublin, Chodzki 4a, Lublin, 20-093 Poland

**Keywords:** dual targeted therapy, multitarget, polypharmacology, polypharmacy, privileged ligands, target repurposing

## Abstract

Drugs with multiple targets, often annotated as ‘unselective’, ‘promiscuous’, ‘multitarget’, or ‘polypharmacological’, are widely considered in both academic and industrial research as a high risk due to the likelihood of adverse effects. However, retrospective analyses have shown that particularly approved drugs bear rich polypharmacological profiles. This raises the question whether our perception of the specificity paradigm (‘one drug-one target concept’) is correct – and if specifically multitarget drugs should be developed instead of being rejected. These questions provoke a paradigm shift – regarding the development of polypharmacological drugs not as a ‘waste of investment’, but acknowledging the existence of a ‘lack of investment’. This perspective provides an insight into modern drug development highlighting latest drug candidates that have not been assessed in a broader polypharmacology-based context elsewhere embedded in a historic framework of classical and modern approved multitarget drugs. The article shall be an inspiration to the scientific community to re-consider current standards, and more, to evolve to a better understanding of polypharmacology from a challenge to an opportunity.

## Polypharmacology Established in Drug Therapy

Polypharmacology is the research field focusing the drugs and medical applications that have effects on multiple targets. It has grown over the last decades into a distinct approach on how to tackle human disease, and its development in various research fields including structural and chemical biology, pharmaceutical and medicinal chemistry, as well as molecular and clinical pharmacology has shed light on polypharmacology from various angles. Nowadays, several aspects can be identified:

- ‘structural polypharmacology’ (structural commonalities despite phylogenetic distance of target proteins)
- ‘molecular polypharmacology’ (molecular-structural motifs that define the multitargeticity of drugs)
- ‘evolutionary polypharmacology’ (structural and functional commonalities between orthologs of different species)
- ‘functional polypharmacology’ [differential mode(s)-of-action of multitarget drugs with multiple targets based on their particular molecular interaction]
- ‘clinical polypharmacology’ (engagement of multiple targets in a therapeutic setting)

**Fig. 1:**
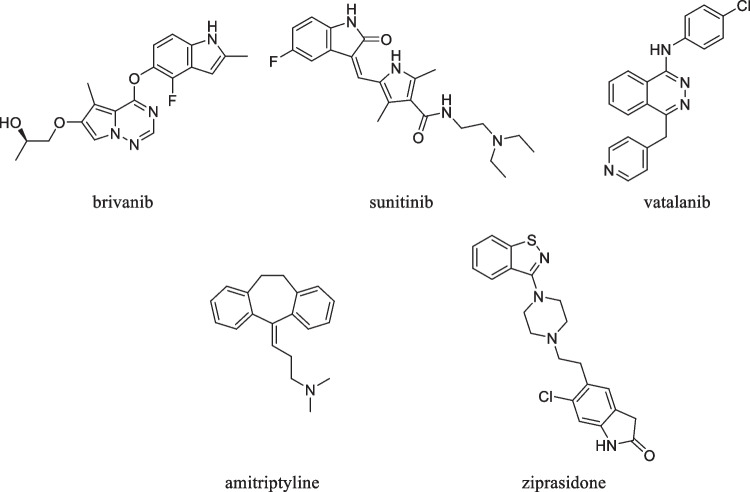
Molecular formulae of approved drugs with polypharmacological profiles.

The ‘polypharmacolome’ is the opportunity space between the above-named aspects in which polypharmacological drugs of the future can be developed by medicinal chemists [1, 2]. Thus, ‘medicinal polypharmacology’ represents an additional part of polypharmacology stretching into other important subfields. However, it still appears to be a theoretical concept rather than an established part of today’s research activities in drug development. The specificity paradigm (‘one drug-one target concept’) has manifested itself as the golden standard for many decades, thereby hindering sincere consideration of alternative approaches. Nevertheless, polypharmacology made its way into clinical application, as demonstrated with two examples from different therapeutic areas:

(i) The development of tyrosine kinase inhibitors (TKIs) was a milestone in advanced anti-cancer therapy around two decades ago. As a matter of fact, the vast majority of TKIs are multitarget drugs that simultaneously target not only one particular (receptor) tyrosine kinase but multiple ones [3]. Three prominent examples are brivanib [inhibiting fibroblast growth factor (FGF) receptors and vascular endothelial growth factor receptors (VEGFRs)], sunitinib [inhibiting colony stimulating factor 1 receptor (CSF1R), FMS-like tyrosine kinase 3 (FLT3), tyrosine-protein kinase KIT (KIT) and tyrosine-protein kinase RET (RET), platelet-derived growth factor receptors α and β (PDGFRA and B), and VEGFR1–3], and vatalanib (inhibiting KIT, PDGFR, and VEGFR) [4–6];
(ii) A much older class of compounds that is genuinely multitargeting is central nervous system-(CNS)-active drugs against psychiatric disorders [7]. These include, for instance, first generation antidepressants such as the tricyclic antidepressant amitriptyline [8–10] or neuroleptics such as ziprasidone [5, 8, 11, 12]. Both drugs inhibit, to a different extent, adrenergic (αR), dopamine (DR), histamine (HR), muscarinic acetylcholine (mAChR), and serotonin receptors (5-HTR) as well as the solute carriers (SLCs) of the SLC6A subfamily that transport dopamine (DAT), noradrenaline (NAT), and serotonin (SERT). Figure 1 shows the molecular formulae of the aforementioned drugs and their molecular-structural diversity.

Interestingly, the above-named drugs stretch with their bioactivity also into other protein superfamilies. The TKIs brivanib [13, 14], sunitinib [13, 15–17], and vatalanib [15, 18] showed both direct inhibition of ABC transporters and/or efficacy against ABC transporters-expressing cancer cell lines. Additionally, sunitinib demonstrated inhibition against the SLC organic cation transporter 1 (OCT1; *SLC22A1*) [19]. Amitriptyline and ziprasidone, on the other hand, had not only an extended polypharmacological profile against SLC transporters [20, 21], but were also reported to interfere with human [21, 22], murine [23, 24], and bacterial [25] ABC transporters as well. These findings suggest that multitarget agents do not only translate between phylogenetically distant human protein families, but also between species. The translation between species is an important approach in ‘target repurposing’ strategies to discover novel antibiotics [26, 27].

## The Immense Potential of Medicinal Polypharmacology

These examples emphasize the tremendous impact that medicinal polypharmacology as the intentional rational development of polypharmacological drugs can have. Clinical indications for which no satisfactory drug therapy is as yet available will benefit in particular from a completely novel drug development approach. In fact, medicinal polypharmacology is advancing in various fields of medical sciences, such as cardiovascular, infectious, malignant, metabolic, neurologic, and neurodegenerative diseases [1, 4, 5, 8, 28, 29].

Interestingly, within the landscape of known small-molecule compounds, drugs by their very nature are comparatively often shown to have polypharmacological profiles [30–32]. One obvious explanation is that drugs were from the very start of both phenotypic and target-based *in vitro* assays the first choice for probing small-molecules toward novel targets / diseases. Thus, drugs were used more often in screenings against other targets than compounds developed for a specific target protein (family). In particular, the latter group of compounds is barely evaluated beyond their distinct target (family) of interest. Another explanation for the large fraction of multitarget agents among approved drugs could also be that the drug evaluation and selection process in clinical trials favors multitarget drugs due to their superior clinical efficacy [30].

While this is only a speculation, it is certainly possible that the clinical picture involves more contributors than the single target at which the drug showed efficacy *in vitro* during the early stage of drug discovery. In other words, only because a possible mode-of-action for a new drug has been identified *in vitro*, it does not necessarily mean that this is the sole mechanism by which it exhibits its therapeutic benefit. Conversely, one could also speculate that these extended polypharmacological profiles of clinical drug candidates, which simultaneously address a network of targets, are indeed essential to achieve the observed therapeutic benefit in a clinical study. Moreover, that these additional drug targets, such as ABC and SLC transporters, in fact also belong to the primary pharmacological targets. Altogether, these aspects emphasize the immense importance and potential of multitarget drugs in clinical application, and the necessity of widely recognizing this not only in academia but also in the drug developing industry.

## Medicinal Polypharmacology in Today’s Drug Discovery

Academic research in the field of medicinal polypharmacology is rapidly growing. The number of polypharmacology-associated publications has seen an exponential increase. For instance, articles with the search term ‘dual targeted therapy’ rose from just over a hundred annually in the early 2000’s to over 2,700 publications in the last year alone. Similarly, ‘multitarget therapy’ or ‘multitarget drug’ increased from a dozen to almost a thousand publications every year during these two decades while the term ‘polypharmacology’ is now associated with over a hundred publications annually.

**Fig. 2:**
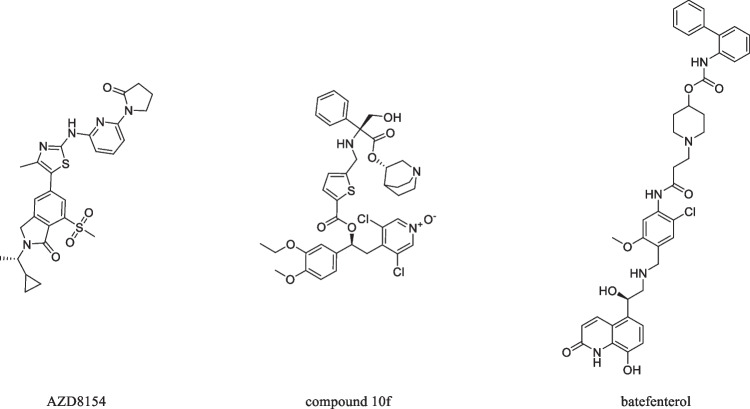
Molecular formulae of polypharmacological drugs and drug candidates.

Expectedly, the majority of these reports were published by academic research groups, however, promising initiatives by a number of pharmaceutical companies emerged that have also realized the potential of polypharmacology. Three notable examples are in the field of pulmonary disorders:

(i) For the treatment of *asthma bronchiale*, AstraZeneca recently reported the development of a drug candidate with physicochemical properties suitable for an inhalative drug application that inhibits two different subtypes of phosphoinositol 3-kinases (PI3K) in leucocytes [33]. AZD8154 (Fig. 2), as it is referred to, was the result of the intentional combination of the activities of the two series of selective PI3Kγ and PI3Kδ inhibitors that had previously been developed by AstraZeneca. It was shown to be safe in a randomized phase I clinical trial, with no reports of serious adverse events, and exhibited pharmacokinetic properties suitable for once-daily drug application [34]. However, a subsequent phase II clinical trial has been withdrawn due to “emerging pre-clinical toxicology findings” (www.clinicaltrials.gov, #NCT04187508);
(ii) For the treatment of chronic obstructive pulmonary disease (COPD), the Italian company Chiesi Farmaceutici in collaboration with Charles River Laboratories very recently combined antagonism at the M_3_ mAChR with inhibition of phosphodiesterase 4 (PDE4) in a so-called MAPI drug candidate (compound 10f; Fig. 2) that showed a balanced bronchodilator / anti-inflammatory profile in rats [35].
(iii) The California-based company Theravance Biopharma, on the other hand, published a few years earlier a so-called MABA*, i.e.*, a muscarinic antagonist and β_2_ adrenergic receptor agonist [36]. Both modes-of-action have been regularly employed in the treatment of COPD using single-mode drugs that demonstrated synergistic effects when given in combination. Theravance Biopharma sought to combine both mechanisms in a single drug, called batefenterol (also referred to as TD-5959 and GSK961081; Fig. 2). Together with GlaxoSmithKline, a number of clinical studies have been conducted to investigate the safety, pharmacokinetics, drug-drug interactions, and beneficial combinations, including a very recent phase II study in combination with a glucocorticoid [37].

**Fig. 3:**
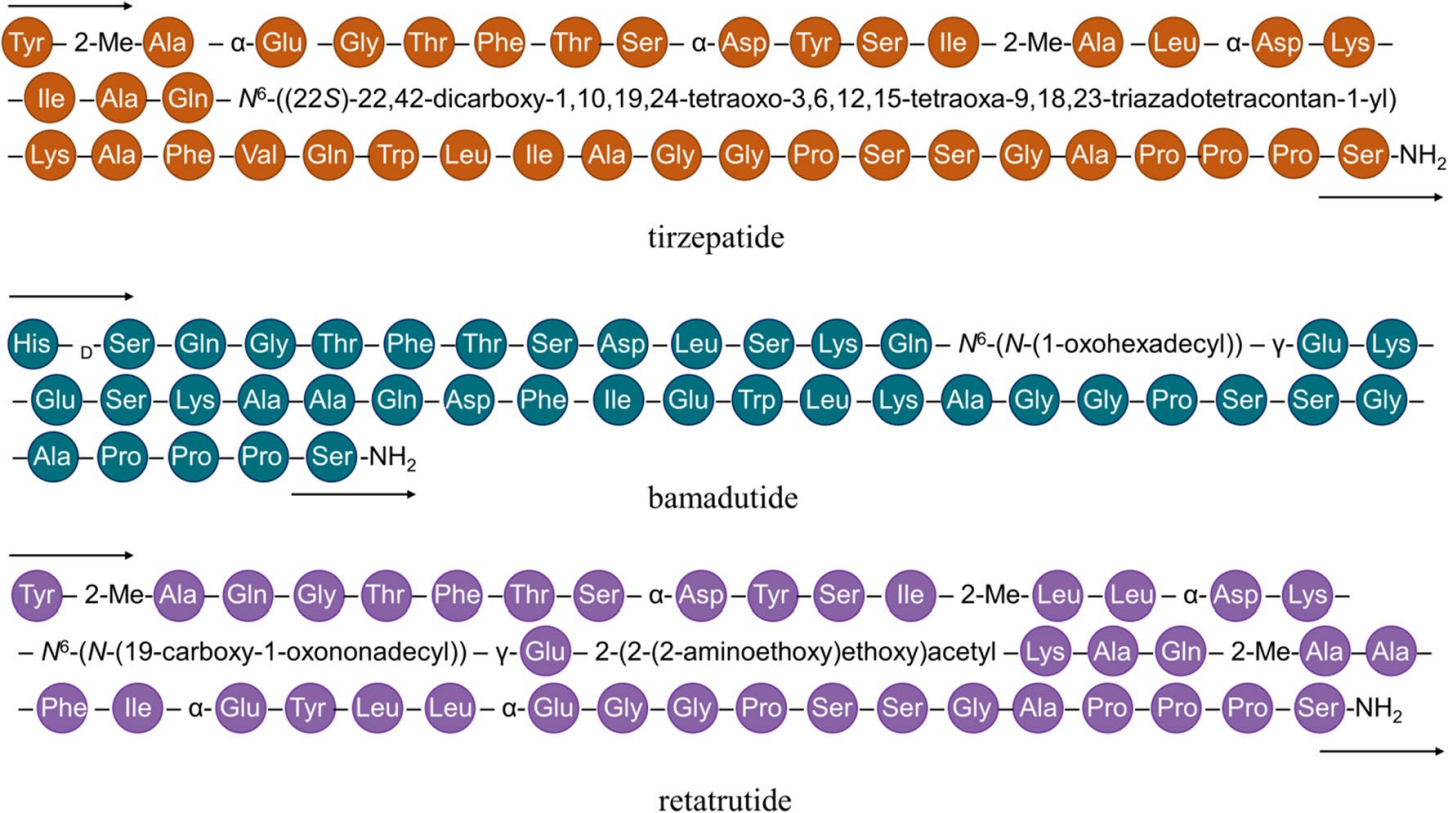
Amino acid sequences of the polypharmacology-by-design drugs tirzepatide, bamadutide, and retatrutide.

Another set of clinical indications for which dual- or even triple-target drugs are currently under development is type 2 *diabetes mellitus*, obesity, and comorbidities:

(i) Tirzepatide (formerly referred to as LY3298176; Fig. 3), a fatty acid modified polypeptide developed by Eli Lilly, is a dual agonist at the receptors for the enteroendocrine incretin hormones glucagon-like peptide 1 (GLP-1) and glucose-dependent insulinotropic polypeptide (GIP) [38]. With approved drugs like exenatide, liraglutide, and semaglutide, selective GLP-1 receptor agonism is currently second-line therapy after metformin. However, gastrointestinal adverse effects prevent dosage increase and thereby higher therapeutic efficacy, especially concerning weight loss. Combining GLP-1 and GIP receptor agonism in a single drug was thus considered to be a possible solution, and this was facilitated by the high degree of sequence similarity between GLP-1 and GIP. Tirzepatide exhibited greater efficacy than selective GLP-1 receptor agonists in several clinical trials as well as a Cochrane meta-analysis [39], and consequently, received approval last year as a first-in-class medication.
(ii) Another approach to ameliorate the established therapeutic benefit of GLP-1 agonism through polypharmacology is the combination with glucagon receptor agonism. Sanofi-Aventis’ drug candidate bamadutide (SAR425899; Fig. 3) successfully passed a number of phase I and one phase II clinical trials [40]. However, another placebo-controlled study evaluating its effects in non-alcoholic fatty liver disease was prematurely terminated due to reasons other than safety concerns (www.clinicaltrials.gov, #NCT034377209, and no further clinical trials have been commenced since.
(iii) Eli Lilly took it a step further and developed the triple-target drug candidate retatrutide (LY3437943; Fig. 3) that showed agonism at the GLP-1, GIP, and glucagon receptors. It also passed a number of phase I and phase II clinical trials [41–43]. Several phase III studies on patients suffering from type 2 *diabetes mellitus* and/or obesity with and without cardiovascular disease, osteoarthritis, or chronic kidney disease are currently being conducted (clinicaltrials.gov, accessed October 20, 2023). Thus, retatrutide might very well become another success story of medicinal polypharmacology.

## Concluding Remarks

In summary, polypharmacology has always been an integral part of drug therapy, although unintentional and unknowingly until this concept has first been formulated two decades ago. The examples discussed here emphasize the great potential of multitarget drugs and delineate the impact they can have on patients suffering from complex diseases for which the specificity paradigm failed to provide adequate solutions. Nevertheless, deliberate medicinal polypharmacology is still rather an exception than the norm in current drug development. A more widespread awareness and sincere appreciation of polypharmacology and its benefits, especially in the pharmaceutical industry, are crucial to harness its full potential toward more effective drug therapies.

## Data Availability

This article contains no datasets generated or analyzed during the current study.
